# miR-377 induces senescence in human skin fibroblasts by targeting DNA methyltransferase 1

**DOI:** 10.1038/cddis.2017.75

**Published:** 2017-03-09

**Authors:** Hong-fu Xie, Ying-zi Liu, Rui Du, Ben Wang, Meng-ting Chen, Yi-ya Zhang, Zhi-li Deng, Ji Li

**Affiliations:** 1 Department of Dermatology, Xiangya Hospital, Central South University, Changsha, China; 2 Center for Molecular Medicine, Xiangya Hospital, Central South University, Changsha, China; 3 State Key Laboratory of Medical Genetics, Central South University, Changsha, Hunan, China; 4 Key Laboratory of Organ injury, Ageing and Regenerative Medicine of Hunan Province, Changsha, China

## Abstract

Skin aging is a complicated physiological process and epigenetic feature, including microRNA-mediated regulation and DNA methylation, have been shown to contribute to this process. DNA methylation is regulated by DNA methyltransferase, of which DNA methyltransferase 1 (DNMT1) is the most abundantly known. But evidence supporting its role in skin aging remains scarce, and no report regards its specifical upstream-regulating molecules in the process of skin aging so far. Here, we found that DNMT1 expression was markedly higher in young human skin fibroblasts (HSFs) than that in passage-aged HSFs, and DNMT1 knockdown significantly induced the senescence phenotype in young HSFs. We predicted the upstream miRNAs which could regulate DNMT1 with miRNA databases and found *miR-377* had high homology with a sequence in the 3′-UTR of human DNMT1 mRNA. We confirmed that *miR-377* was a potential regulator of DNMT1 by luciferase reporter assays. *miR-377* expression in passage-aged HSFs was markedly higher than that in the young HSFs. *miR-377* overexpression promoted senescence in young HSFs, and inhibition of *miR-377* reduced senescence in passage-aged HSFs. Moreover, these functions were mediated by targeting DNMT1. Microfluidic PCR and next-generation bisulfite sequencing of 24 senescent-associated genes' promoters revealed alterations of the promoter methylation levels of *FoxD3,* *p53,* and *UTF1* in HSFs treated with *miR-377* mimics or inhibitors. We also verified that the *miR-377*-mediated changes in p53 expression could be reversed by regulation of DNMT1 in HSFs. Similarly, there was a negative correlation between *miR-377* and DNMT1 expression in young and photoaged HSFs, HSFs, or skin tissues from UV-unexposed areas of different aged donors. Our results highlight a novel role for *miR-377*-DNMT1-p53 axis in HSF senescence. These findings shed new light on the mechanisms of skin aging and identify future opportunities for its therapeutic prevention.

Aging is a complicated physiological process that occurs in all organisms. The aging process includes structure retrogression, dysfunction, adaptability, and reduced resistance.^1^ Skin aging is the most obvious sign of organism aging. In aging skin, physiological changes, including structural transformation, biochemical abnormalities, decreased repair capacity, and impaired permeability, appear gradually.^1^ Aged skin exhibits increased wrinkling, sagging, and decreased elasticity mainly because of impairment of connective tissue,^2^ an important component of the dermis. Fibroblasts are the main cells in the dermis and are primarily responsible for synthesizing collagen and extracellular matrix to maintain the structural integrity of the skin.^3^ During the process of skin aging, various biological changes occur in fibroblasts, including cell cycle abnormalities, DNA damage, apoptosis, and extracellular matrix synthesis and degradation, affecting the condition and function of the skin. Therefore, fibroblast senescence may play an important role in skin aging. The mechanisms through which senescence is induced in skin fibroblasts are complicated and include excessive oxidative stress,^4^ impaired mitochondria,^5^ deficient immune function,^6^ and alterations in genetic factors. Epigenetic alterations, for example, DNA methylation, histone modifications, and chromatin remodeling, are also thought to be critical in aging.^7^ However, the roles of epigenetic alterations in the senescence of skin fibroblasts are largely not known.

DNA methyltransferase 1 (DNMT1), which was first identified in 1988 by Bestor,^8^ primarily functions to maintain the methylated state after DNA replication and to ensure that this methylation state was passed on to the offspring cells. High-throughput analysis revealed the changes of global genome methylation levels and patterns during skin aging.^9, 10, 11, 12^ While roles and mechanisms of DNA methyltransferase in skin aging was rare, *Qian H, et al.* discovered that the expression of Dnmt3a, Dnmt3b, and Tet2 declined significantly in mouse skin during ageing.^13^ Notably, progressive alopecia appeared during aging of mice with epidermal loss of DNMT1, which was related to defeat in stem cell homeostasis maintaining.^14^ In addition, we found in our preliminary experiment that epidermis-specific DNMT1 knockdown in mice resulted in premature aging-like phenotypes, such as pachylosis, alopecia, and deep wrinkles (data not shown). So, we conferred DNMT1 might play a vital role in cellular senescence and skin aging. Nevertheless, its function in dermal fibroblast senescence remains unclear. Because of the important roles of DNMT1 in aging and other cellular processes, it will be important to elucidate the mechanisms that regulate the expression, stability, and activity of DNMT1, including transcriptional regulation, post-transcriptional auto-inhibitory controls, and post-translational modifications.^15^ The transcriptional promotion of DNMT1 gene expression is induced by signal transducer and activator of transcription 3 in malignant T cells,^16^ estrogen receptor *α* in human breast cancer MCF-7 cells,^17^ and Oct4 and Nanog in MSCs.^18^ HMG-box transcription factor 1 has been reported to be a transcriptional repressor of the *DNMT1* gene in 2BS and WI-38 cells.^19^ Moreover, in zebrafish hepatocytes, overexpressing ubiquitin-like with PHD and ring finger domains 1 results in delocalization and destablization of DNMT1.^20^ Post-translational modifications, including acetylation, ubiquitination, phosphorylation, and methylation, regulate the stability of DNMT1 protein.^21^ Furthermore, various microRNAs (miRNAs),^22^ such as *miR-29*, *miR-140*, *miR-148*, *miR-152*, and *miR-185*, have been reported to target and modulate DNMT1 in various cancer cells. However, no studies have examined the roles of miRNAs in regulating DNMT1 in the context of cellular senescence or skin aging. In our preliminary work, we used computational miRNA target analysis from a miRNA database to identify the miRNA target sites of the 3′-untranslated region (UTR) of human DNMT1 mRNA and identified *miR-377* as a DNMT1 regulator. *miR-377* has been reported to enhance fibronectin protein production,^23^ regulate angiogenesis,^24^ suppress cell proliferation,^25, 26^ predict clinical outcomes in patients with gastric cancer, induce tumorigenesis,^27^ and promote oxidative stress.^28^ Owing to the pleiotropic functions and DNMT1 targeting potential of *miR-377*, *miR-377* may regulate human skin fibroblast (HSF) senescence by targeting DNMT1.

Thus, in this study, we examined whether *miR-377* and DNMT1 were important molecules and *miR-377* could directly target and inhibit DNMT1 during HSF senescence. We also explored the downstream effects of *miR-377*-dependent regulation of DNMT1, including *p53* methylation and HSF senescence. Our data provided evidence for the role of the *miR-377*-DNMT1-p53 axis in HSF senescence.

## Results

### Expression and function of DNMT1 in HSF senescence

**Figure 1:**
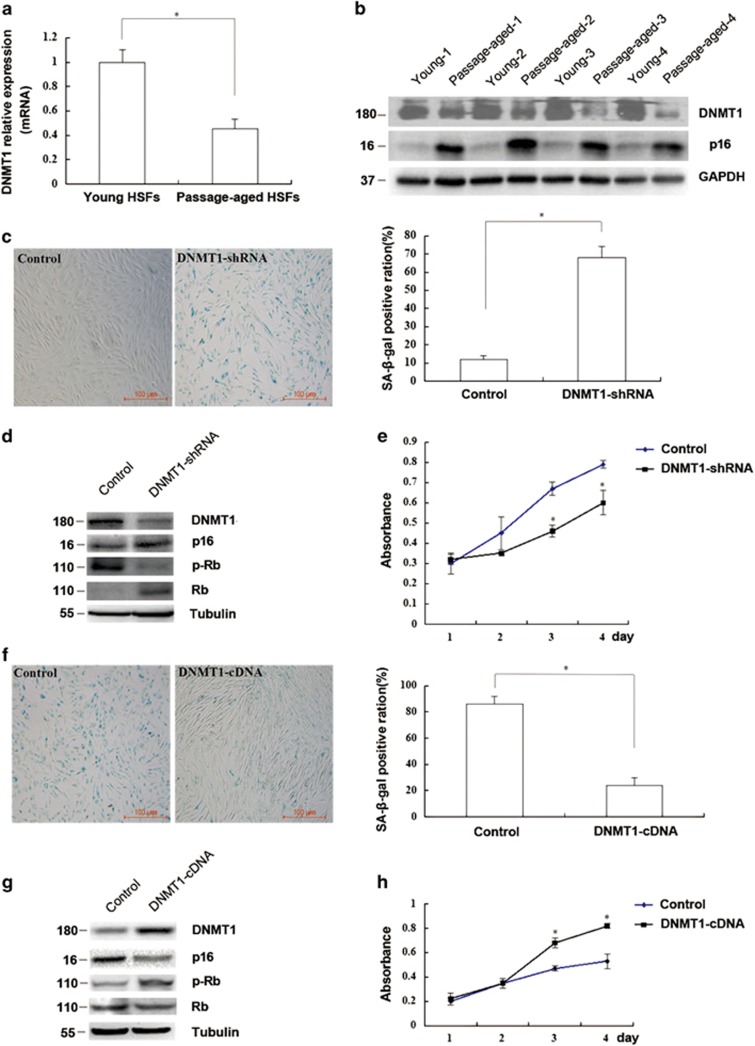
The expression and role of DNMT1 in HSFs senescence. (**a**) DNMT1 mRNA in passage-aged (PD>50) and young (PD<10) HSFs was detected by RT-qPCR (Data represented as the mean±S.E.M. *n*=10, *P<0.05). Representative data was shown. (**b**) DNMT1 and p16 protein levels in passage-aged (PD>50) and young (PD<10) HSFs were detected by western blot (*n*=10, *P<0.05). Representative data was shown. (**c**) SA-*β*-gal-positive cells in young HSFs (PD<10) transfected with control shRNA or DNMT1-shRNA were detected by using kit (left). The SA-*β*-gal-positive ratio was shown (right; Data represented as the mean±S.E.M. *n*=3, *P<0.05). (**d**) DNMT1, p16, and Rb protein expressions and phosphorylation of Rb in young HSFs (PD<10) transfected with control shRNA or DNMT1-shRNA were detected by western blot (*P<0.05). Representative data was shown. (**e**) Absorbance at 490 nm in young HSFs (PD<10) transfected with control shRNA or DNMT1-shRNA was detected by MTS assays (Data represented as the mean±S.E.M. *n*=3 at each time point, *P<0.05). (**f**) SA-*β*-gal-positive cells in the passage-aged HSFs (PD>50) transfected with control cDNA or DNMT1 cDNA were detected by using kit (left). The SA-*β*-gal-positive ratio was shown (right; data represented as the mean±S.E.M. *n*=3, *P<0.05). (**g**) DNMT1, p16, and Rb protein expressions and phosphorylation of Rb were detected by western blot in the passage-aged HSFs (PD>50) after being transfected with control cDNA or DNMT1 cDNA (*P<0.05). Representative data was shown. (**h**) Absorbance at 490 nm was detected in the passage-aged HSFs (PD>50) after being transfected with control cDNA or DNMT1 cDNA by MTS assays. (Data represented as the mean±S.E.M. *n*=3 at each time point, *P<0.05)

First, we analyzed the expression of DNMT1 in ten pairs of young (population doubling (PD) <10) and passage-aged (PD>50) HSFs by real time-quantitative PCR (RT-qPCR) and western blotting. DNMT1 expression in passage-aged HSFs (PD>50) with higher p16 expression was markedly lower than that in young HSFs (PD<10) at both the mRNA and protein levels (*P*<0.05; Figures 1a and b). In addition, DNMT1 knockdown increased the senescence-associated *β*-galactosidase (SA-*β*-gal) staining rate, p16 and Rb (Restinoblastoma tumor suppressor protein) expressions while reduced the phosphorylation level of Rb, and cell proliferation in young HSFs (PD<10) compared with that in the control group (*P*<0.05; Figures 1c–e). Besides, two DNMT1-shRNA (DNMT1-shRNA and DNMT-shRNA′) sequences were applied to eliminate the chance of off-target in gene silencing, while no alterations of DNMT(3a,3b) expression were detected in DNMT1 knockdown, which excluded the possibility that *DNMT1* gene silencing may affect other DNMTs (Supplementary Figure S1). Inversely, upregulation of DNMT1 in passage-aged HSFs (PD>50) reduced the SA-*β*-gal staining rate, p16 and Rb expressions while increased the phosphorylation level of Rb and cell proliferation compared with that in the control group (*P*<0.05; Figures 1f–h). Taken together, these results suggested that DNMT1 may play an important role in the process of HSFs senescence.

### miR-377 regulated DNMT1 expression by directly targeting DNMT1 in HSFs

**Figure 2:**
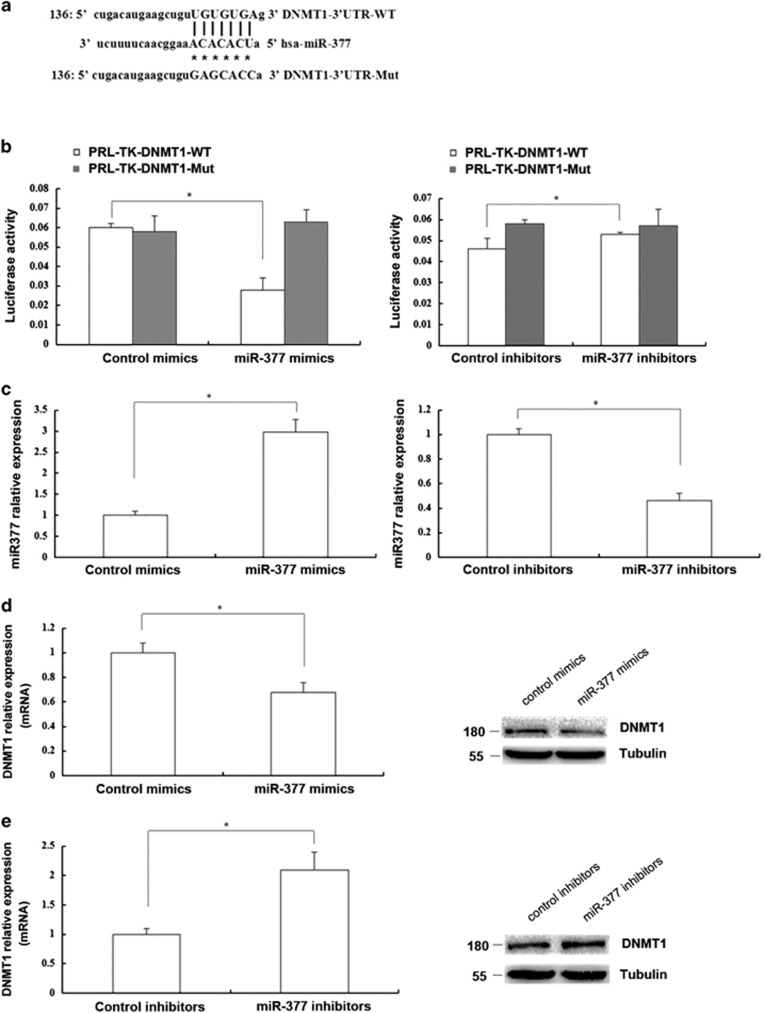
*miR-377* could regulate DNMT1 expression by directly targeting DNMT1 in HSFs. (**a**) Though bioinformatics prediction, the sequence of the *miR-377* binding site in the 3′-UTR of DNMT1 was shown at the upper site. Mutated residues were shown at the lower site. (**b**) Luciferase activity change of the wild-type 3′-UTR reporters and the mutant 3′-UTR reporters in 293T cells treated with control mimics or *miR-377* mimics (left) and 293T cells treated with control inhibitors or miR-377 inhibitors (right) was shown, respectively (Data represented as the mean±S.E.M. *n*=3, *P<0.05, respectively). (**c**) *miR-377* level in young HSFs (PD<10) treated with control mimics or miR-377 mimics (left) and in passage-aged HSFs (PD>50) treated with control inhibitors or *miR-377* inhibitors (right) was respectively detected by RT-qPCR (Data represented as the mean±S.E.M. *n*=3, *P<0.05, respectively). (**d**) DNMT1 mRNA and protein expression in the young HSFs (PD<10) treated with control mimics or *miR-377* mimics was detected by RT-qPCR and western blot, respectively (Data represent the mean±S.E.M. *n*=3, *P<0.05). (**e**) DNMT1 mRNA and protein expression in the passage-aged HSFs (PD>50) treated with control inhibitors or *miR-377* inhibitors was detected by RT-qPCR and western blot, respectively (Data represent the mean±S.E.M. *n*=3, *P<0.05)

Computational miRNA target analysis from a miRNA database demonstrated that *miR-377* had high homology with a sequence in the 3′-UTR of human DNMT1 mRNA (Figure 2a). To confirm whether *miR-377* directly target DNMT1, we constructed a wild-type (WT) DNMT1 3′-UTR luciferase reporter vector and a *miR-377* homologous sequence mutant DNMT1 3′-UTR luciferase reporter vector. Expression of *miR-377* mimics decreased the relative luciferase activity of the wild-type reporter (*P*<0.05) but had no effect on the mutant reporter compared with the control in HEK293T cells (*P*>0.05). Moreover, expression of *miR-377* inhibitors increased the relative luciferase activity of the wild-type reporter (*P*<0.05) but did not affect that of the mutant reporter compared with the control in HEK293T cells (*P*>0.05; Figure 2b).

Next, we examined the effects of *miR-377* on the expression of DNMT1 in HSFs. We treated HSFs with *miR-377* mimics or inhibitors and measured the DNMT1 expression (Figure 2c). DNMT1 mRNA and protein expression levels were significantly decreased by the *miR-377* mimics (*P*<0.05; Figure 2d) and elevated by the *miR-377* inhibitors (*P*<0.05; Figure 2e). Collectively, our findings indicated that *miR-377* suppressed DNMT1 expression by directly targeting DNMT1 in HSFs.

### miR-377 mediated senescence in HSFs

**Figure 3:**
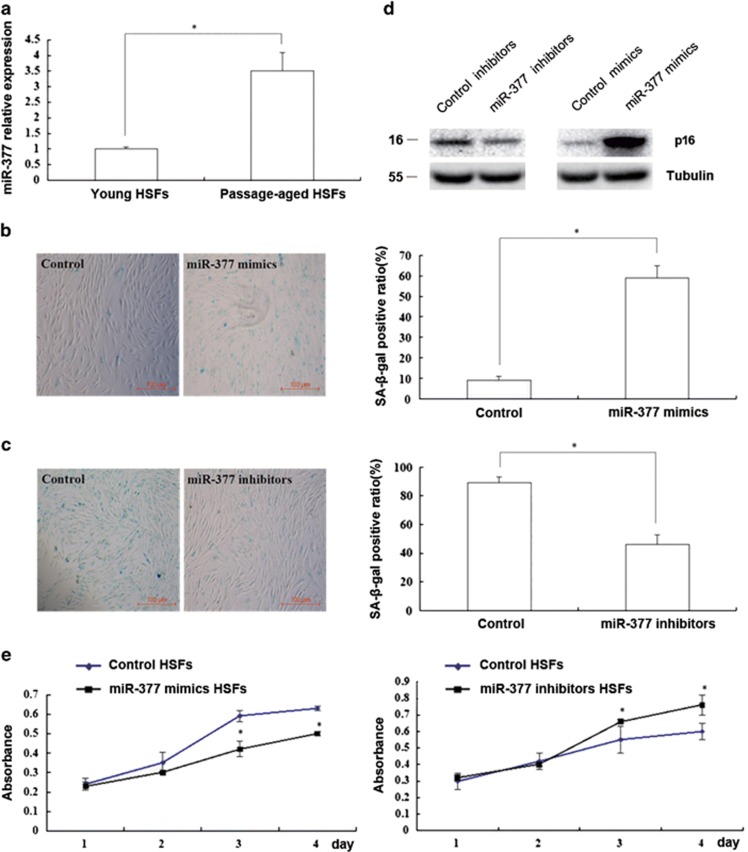
*miR-377* mediated senescence in HSFs. (**a**) *miR-377* level in the young (PD<10) and passage-aged (PD>50) HSFs was detected by RT-qPCR (Data represented as the mean±S.E.M. *n*=10, *P<0.05). (**b**) SA-*β*-gal-positive cells in young HSFs (PD<10) treated with control mimics or miR-377 mimics were detected by using kit (left). The SA-*β*-gal-positive ratio was shown (right; data represented as the mean±S.E.M. *n*=3, *P<0.05). (**c**) SA-*β*-gal-positive cells in passage-aged HSFs (PD>50) treated with control inhibitors or miR-377 inhibitors were detected by using kit (left). The SA-*β*-gal-positive ratio was shown (right; data represented as the mean±S.E.M. *n*=3, *P<0.05). (**d**) p16 protein expression was respectively detected by western blot in passage-aged HSFs (PD>50) treated with control inhibitors or miR-377 inhibitors (left) and in young HSFs treated with control mimics or miR-377 mimics (right; *n*=3, *P<0.05). Representative data was shown. (**e**) Absorbance at 490 nm was respectively detected by MTS assays in young HSFs (PD<10) treated with control mimics or miR-377 mimics (left) and in passage-aged HSFs treated with control inhibitors or miR-377 inhibitors (right; data represented as the mean±S.E.M. *n*=3 at each time point, *P<0.05)

To investigate the role of *miR-377* in HSF senescence, we analyzed the expression of *miR-377* in HSFs. We found that *miR-377* expression was significantly higher in passage-aged HSFs (PD>50) than in the young HSFs (PD<10; *P*<0.05; Figure 3a). Moreover, overexpression of *miR-377* increased the SA-*β*-gal-positive ratio and p16 expression while decreased HSF proliferation in young HSFs (PD<10; *P*<0.05; Figures 3b, d and e). In contrast, inhibition of *miR-377* reduced the SA-*β*-gal-positive ratio and p16 expression while increased cell proliferation in passage-aged HSFs (PD>50; *P*<0.05; Figures 3c–e). Taken together, our data indicated that *miR-377* played an important role in HSF senescence.

### miR-377 promoted senescence by suppressing DNMT1 expression in HSFs

**Figure 4:**
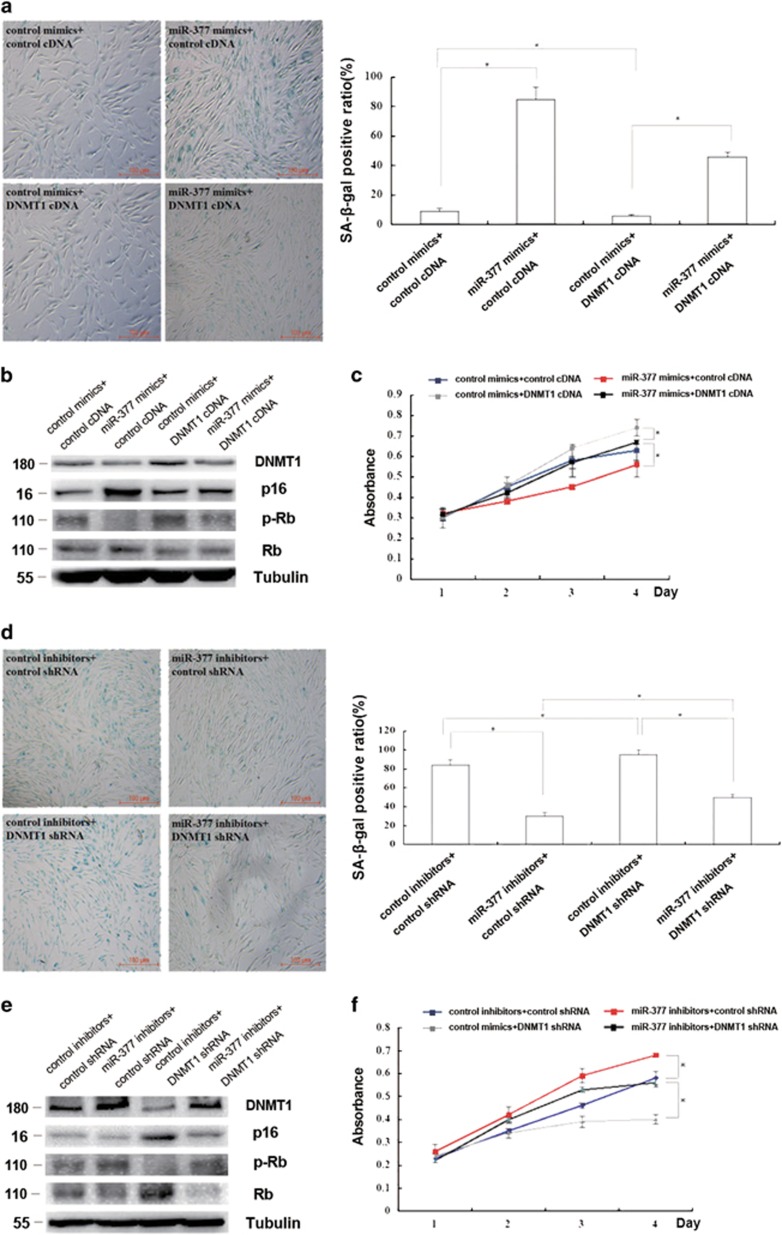
*miR-377* promoted senescence by suppressing DNMT1 expression in HSFs. (**a**) Cellular senescence was detected by evaluating SA-*β*-gal-positive cells in young HSFs (PD<10) treated with miR-377 mimics together with control cDNA or DNMT1 cDNA as indicated (left). The positive cell quantification was shown (right; data represent the mean±S.E.M. *n*=3, *P<0.05). (**b**) DNMT1, p16, and Rb expressions and phosphorylation level of Rb were detected by western blot in young HSFs (PD<10) treated with miR-377 mimics together with control cDNA or DNMT1 cDNA as indicated (*P<0.05). Representative data was shown. (**c**) Absorbance at 490 nm was detected by MTS assays in young HSFs (PD<10) treated with miR-377 mimics together with control cDNA or DNMT1 cDNA as indicated (data represented as the mean±S.E.M. *n*=3 at each time point, *P<0.05). (**d**) Cellular senescence was detected by evaluating SA-*β*-gal-positive cells in passage-aged HSFs (PD>50) treated with *miR-377* inhibitors together with control shRNA or DNMT1 shRNA as indicated (left). The SA-*β*-gal-positive ratio was shown (right; data represented as the mean±S.E.M. *n*=3, *P<0.05). (**e**) DNMT1, p16, and Rb expressions and phosphorylation level of Rb were detected by western blot in passage-aged HSFs (PD>50) treated with miR-377 inhibitors together with control cDNA or DNMT1 cDNA as indicated (*P<0.05). Representative data was shown. (**f**) Absorbance at 490 nm was detected by MTS assays in passage-aged HSFs (PD>50) treated with miR-377 inhibitors together with control cDNA or DNMT1 cDNA as indicated (data represented as the mean±S.E.M. *n*=3 at each time point, *P<0.05)

To examine how *miR-377* exerts its functions, we altered DNMT1 expression in HSFs with miR-377 overexpressed or knocked-down. Enhancement of DNMT1 expression partly blocked the increased SA-*β*-gal activity, p16 and Rb levels together with the decreased phosphorylation of Rb and the proliferation in young HSFs (PD<10) overexpressing *miR-377* (*P*<0.05; Figures 4a–c). Conversely, suppression of DNMT1 expression partly restored the decreased SA-*β*-gal activity, p16 and Rb levels together with the increased phosphorylation of Rb and the proliferation in passage-aged HSFs (PD>50) in the presence of *miR-377* inhibitors (*P*<0.05; Figures 4d–f). Collectively, these findings indicated that *miR-377* regulated HSF senescence, potentially through modulation of DNMT1 expression.

### Role of miR-377 in modulating promoter methylation levels of senescent-associated genes in HSFs

**Figure 5:**
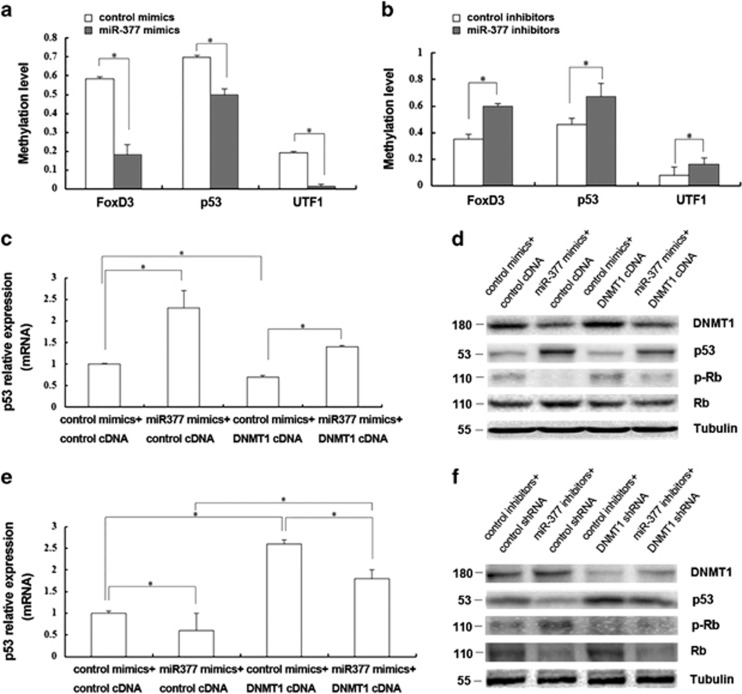
Role of *miR-377* in modulating promoter methylation levels of senescent-associated genes in HSFs. (**a**) The promoter methylation levels of *FoxD3, p53* and *UTF1* were analyzed in young HSFs (PD<10) transfected with control mimics or miR-377 mimics through microfluidic PCR and next-generation bisulfite sequencing (data represented as the mean±S.E.M. *P<0.05). (**b**) The promoter methylation levels of *FoxD3, p53* and *UTF1* were analyzed in passage-aged HSFs (PD>50) transfected with control inhibitors or *miR-377* inhibitors through microfluidic PCR and next-generation bisulfite sequencing (data represented as the mean±S.E.M. *P<0.05). (**c**) p53 mRNA was detected by RT-qPCR in young HSFs (PD<10) treated with *miR-377* mimics together with control cDNA or DNMT1 cDNA as indicated (data represented as the mean±S.E.M. *n*=3, *P<0.05). (**d**) DNMT1, p53, and Rb expressions and phosphorylation of Rb were detected by western blot in young HSFs (PD<10) treated with miR-377 mimics together with control cDNA or DNMT1 cDNA as indicated (*P<0.05). Representative data was shown. (**e**) p53 mRNA was detected by RT-qPCR in passage-aged HSFs (PD>50) treated with miR-377 inhibitors together with control cDNA or DNMT1 cDNA as indicated (data represented as the mean±S.E.M. *n*=3, *P<0.05). (**f**) DNMT1, p53, and Rb expressions and phosphorylation of Rb were detected by western blot in passage-aged HSFs (PD>50) treated with miR-377 inhibitors together with control shRNA or DNMT1 shRNA as indicated (*P<0.05). Representative data was shown

DNMT1 functions by methylating DNA, thereby regulating gene expression and other processes. To determine whether *miR-377*-mediated changes in DNMT1 expression influence the methylation of the promoters of senescence-associated genes, we treated HSFs with the *miR-377* mimics or inhibitors and analyzed promoter methylation after 48 h. Promoter methylation levels of 24 senescent-associated genes (Supplementary Table 1) were analyzed through microfluidic PCR and next-generation bisulfite sequencing. From these analyses, the promoters of three genes were found to be significantly methylated (Figures 5a and b). Of these three genes, *p53* was further confirmed to be regulated by *miR-377* using RT-qPCR and western blotting. Increased p53 mRNA, p53 and Rb expressions together with diminished phosphorylation of Rb were induced by the *miR-377* mimics and reverse changes were induced by the *miR-377* inhibitors compared with that of the respective controls (*P*<0.05; Figures 5c–f). Furthermore, modulation of DNMT1 expression could partly reverse the changes in protein levels of p53, Rb and phosphorylation of Rb induced by *miR-377* (Figures 5c–f). These data suggested that *miR-377* could regulate the methylation of the p53 promoter and thus influence HSF senescence through modulation of DNMT1 expression.

### miR-377 and DNMT1 expression in intrinsic aging of skin *in vivo* and in photoaged HSFs

**Figure 6:**
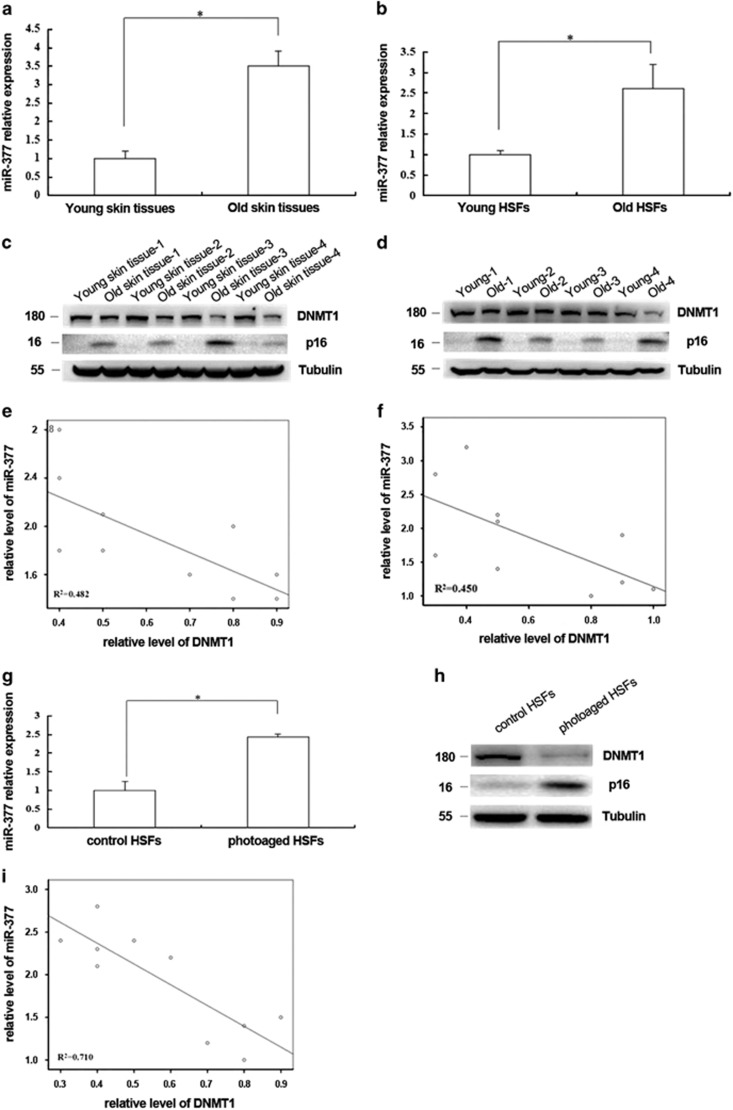
*miR-377* and DNMT1 expression *in vivo* and photoaged HSFs. (**a**) *miR-377* level was detected by RT-qPCR in the young and the old skin tissues. (Data represented as the mean±S.E.M. *n*=10, *P<0.05). (**b**) miR-377 level was detected by RT-qPCR in HSFs from the young and the old skin tissues (Data represented as the mean±S.E.M. *n*=10, *P<0.05). (**c**) DNMT1 and p16 expressions were detected by western blot in the young and the old skin tissues (*n*=10, *P<0.05). Representative data was shown. (**d**) DNMT1 and p16 expressions were detected by western blot in HSFs from the young and the old skin tissues (*n*=10, *P<0.05). Representative data was shown. (**e** and **f**) The correlations between *miR-377* and DNMT1 levels in different couples of young and old skin tissues and in HSFs from young and old skin tissues were shown respectively (*n*=10, *R*^2^=0.482,0.450, respectively, *P<0.05). (**g**) *miR-377* level was detected in control and photoaged HSFs by RT-qPCR (Data represented as the mean±S.E.M. *n*=3, *P<0.05). (**h**) DNMT1 and p16 expression levels were detected by western blot in control and photoaged HSFs (*P<0.05). Representative data was shown. (**i**) The correlation between *miR-377* and DNMT1 levels was analyzed in several couples of UVA-untreated and UVA-treated HSFs (*n*=10, *R*^2^=0.471, *P<0.05)

To evaluate the potential roles of *miR-377* and DNMT1 in the intrinsic aging of skin, we examined *miR-377* and DNMT1 expression levels in primary cultured HSFs or skin tissues from ultraviolet (UV)-unexposed areas of differently aged donors (<10 years old and >65 years old) by RT-qPCR and western blotting. The results showed that HSFs and skin tissues from old donors with higher level of p16 expression had significantly higher levels of *miR-377* and lower levels of DNMT1 than those from young donors with lower p16 expression (Figures 6a–d). Negative correlations were identified between *miR-377* and DNMT1 expression in HSFs or skin tissues collected from UV-unexposed areas of differently aged individuals (Figures 6e and f; *R*^2^=0.482, *R*^2^=0.45, respectively; *P*<0.05). In addition, *miR-377* was also increased and DNMT1 was decreased in UVA-induced senescent HSFs (*P*<0.05; Figures 6g and h) and a similar negative correlation was found between *miR-377* and DNMT1 expression in UVA-induced senescent HSFs (Figure 6i, *R*^2^=0.710, *P*<0.05).

## Discussion

In this study, we examined the roles of *miR-377* and DNMT1 in senescence in HSFs. Our results showed that *miR-377* regulated the methylation of *p53* through direct targeting DNMT1, thereby mediating HSF senescence. These findings may have implications in the therapeutic prevention of skin aging.

Skin aging is a complicated physiological process. Older monozygotic twins have been shown to exhibit marked differences in skin aging phenotypes, despite sharing a common genotype; these differences may be explained by distinct profiles of DNA methylation in the twins.^29^ DNA methylation is regulated by DNMTs, of which DNMT1 is the most abundant. Knockdown of DNMT1 by siRNA has been shown to alter the methylation of various CpG islands and induce senescence in human umbilical cord blood-derived stem cells.^30^ Moreover, mutations in DNMT1 can cause both central and peripheral neurodegeneration through aberrant methylation.^31^ Studies on Dnmt1^+/−^ mice have shown that changes in DNA methylation may contribute to some forms of aging-related amyloidosis.^32^ In our present study, we found, for the first time, that DNMT1 expression decreased with age in passage-aged HSFs and that downregulation of DNMT1 aggravated the senescent phenotype in young HSFs. Our data were consistent with previous studies, which indicated that DNMT1 is involved in the process of cellular senescence in the skin.

Many studies have suggested that epigenetic modifications are deeply involved in the modulation of DNMT1, including miRNA regulation.^33, 34^ No reports have described the targeting of DNMT1 by miRNAs in the context of aging. Bioinformatics analysis of the DNMT1 promoter showed that *miR-377*, with 7 bases predicted complementarily pairing with DNMT1 3′UTR, which has been shown to function as a tumor suppressor in various types of cancer cells, including clear cell renal cell carcinoma,^35^ malignant melanoma,^36^ hepatocellular carcinoma,^25^ osteosarcoma,^26^ glioblastoma,^37^ and prostate cancer cells,^38^ may regulate DNMT1. Furthermore, *miR-377* is involved in angiogenesis^24^ through targeting of vascular endothelial growth factor. Interestingly, *miR-377* can promote oxidative stress by suppressing superoxide dismutase (SOD) 1 and SOD2, increasing p38 mitogen-activated protein kinase (MAPK) phosphorylation and thioredoxin-interacting protein expression, and activating the NLR family, pyrin domain-containing 3 (NLRP3) inflammasome pathway,^28^ indicating that *miR-377* can influence important aging-related factors. In our study, we found that *miR-377* expression was significantly increased in passage-aged HSFs and that inhibition of *miR-377* could reverse the passage-induced senescence of HSFs; this effect could be partly rescued by knockdown of DNMT1. We confirmed, for the first time, that *miR-377* acted as a senescence inducer by regulating DNMT1 in HSFs. Moreover, negative correlations were observed between *miR-377* and DNMT1 expression in passage-aged HSFs, photoaged HSFs, and intrinsic aged HSFs and skin tissues in our present study, supporting that *miR-377* and DNMT1 played important roles in various types of cellular senescence and aging processes in the skin.

DNA methylation by DNMT1 is an important epigenetic mechanism that functions to maintain correct gene expression and stability. Dysregulation of DNMT1 is involved in a variety of diseases.^39^ Previous studies have shown that DNMT1 can regulate the methylation level of the PTEN,^40^ p16INK4A,^41^ p53, and p21^42^promoters. p53 is a well-known tumor suppressor that may also mediate the aging process. Knockdown of the Caenorhabditis elegans *p53* gene (Cep-1) can increase lifespan.^43^ Furthermore, an epidemiological study showed that a mutation in the p53 protein (R72P) increases survival and contributes to aging.^44^ Recent data have suggested that p53 is an important inducer of organismal aging; for example, p53-mediated skin aging involves the loss of subdermal fat and declining sebaceous gland activity.^45^ In our study, we found that methylation of the p53 promoter was significantly altered after alterations in *miR-377* expression in HSFs. We further confirmed that *miR-377* could induce p53 expression and that this effect could be reversed by upregulation of DNMT1. Thus, our study provided evidence to support that the *miR-377*-DNMT1-p53 axis plays an important role in HSF senescence. Identification of the roles of *miR-377* and DNMT1 in skin fibroblast senescence would facilitate the identification of potential new targets or drugs to prevent or cure skin aging.

Collectively, our study demonstrates that *miR-377* directly inhibit DNMT1, thereby regulating *p53* methylation and promoting senescence in HSFs. These data provide a new target for the prevention of skin aging. Further study should focus on comprehensive exploration of upstream regulation of DNMT1 and how to regulate *miR-377* during the natural aging of skin.

## Materials and Methods

### Skin tissues and cell culture

UV-unexposed normal skin tissues were collected from normal UV-unexposed areas surrounding the surgical sites from patients with benign dermatosis in the Department of Dermatology, Xiangya Hospital of Central South University. Patients were informed in advance and provided written consent for the collection and use of their tissues for research purposes. This study was conducted in accordance with the principles of the Declaration of Helsinki. Skin tissues from individuals ages 1–10 years old were used as the young group, whereas those from patients ages 65 years and older were used as the old group. Primary HSFs were isolated by digesting human skin with type II collagenase (Sigma-Aldrich, St. Louis, MO, USA), as described previously, and then cultured in Dulbecco's modified Eagle's medium (Thermo Scientific, Waltham, MA, USA) containing 10% fetal bovine serum (Gibco/Thermo Scientific, Waltham, MA, USA), 100 U/ml penicillin, and 100 *μ*g/ml streptomycin at 37 °C in an atmosphere containing 5% CO_2_. The HSFs isolated from the young group were divided into the young HSF group (PD<10) and passage-aged group (PD>50). Similarly, HSFs (PD<10) cultured from the two groups of skin tissues were designated young HSFs and old HSFs, respectively.

### HSF photoaging model

In UVA-induced senescent HSFs, young HSFs at 60% confluence were washed with phosphate-buffered saline (PBS) at 37 °C twice, covered with 3 ml PBS, and treated with 10 J/cm^2^ UVA/day (Philips TL20W/12 RS UVA lamps; American Ultraviolet Company, Murray Hill, NJ, USA) for 3 consecutive days.

### SA-*β*-gal staining

Senescence was determined using a *β*-galactosidase activity staining kit (Cell Signaling Technology, Danvers, MA, USA) following the manufacturer's recommendations. HSFs were cultured until 80% confluence and washed with PBS twice. The cells were fixed at room temperature for 15 min. After fixation, the cells were washed with PBS three times and incubated with freshly prepared *β*-galactosidase staining solution at 37 °C free of CO_2_ overnight. The stained cells were imaged using a microscope, and the aging ratio of the cells was calculated.

### RNA isolation and miRNA quantification

Total RNA from the HSFs was isolated with TRIzol (Thermo Scientific), and cDNA was prepared using a RevertAid First Strand cDNA Synthesis Kit (Gibco/Thermo Scientific). real time-quantitative PCR (RT-qPCR) was performed using the Thermo Scientific miRNA assay protocol with *miR-377* or U6-specific primers (Ribobio Company, Guangzhou, China) with a Bio-Rad real-time PCR system (Bio-Rad, Hercules, CA, USA). The relative expression levels of *miR-377* were calculated with the 2^−ΔΔCt^ method using U6 as the endogenous control.^46^ DNMT1 and GAPDH mRNA levels were determined as described above with GAPDH as the endogenous normalization control. Primers are available on request.

### Transfection

HSFs were transfected with the *miR-377* mimic, *miR-377* inhibitor, or scrambled controls at a final concentration of 20 *μ*M using Lipofectamine reagent (Thermo Scientific, Waltham, MA, USA) according to the manufacturer's instructions. The cells were subsequently incubated at 37 °C for 48 h.

### MTS assay

HSFs were seeded in 96-well culture dishes at 5000 cells/well. Next, 20 *μ*l CellTiter 96 AQueous MTS Reagent (Promega, Madison, WI, USA) was added to each well. The culture dishes were further incubated for 4 h at 37 °C in an atmosphere containing 5% CO_2_ in the dark. The absorbance of the samples was measured at 490 nm. All measurements were repeated three times.

### Western blotting

Western blotting was performed as previously described.^47^ Briefly, the cells were rinsed twice with ice-cold PBS and collected with radioimmunoprecipitation assay buffer. Total protein was quantified, separated on 10% SDS/polyacrylamide gels, and transferred to PVDF membranes. The membranes were blocked in 5% nonfat milk for 1 h at room temperature and probed with specific antibodies or anti-*β*-actin antibodies at 4 °C overnight, followed by incubation with a secondary antibody conjugated to horseradish peroxidase. Immunoreactivity was detected with Super Signal West Pico Chemiluminescent Substrate (Thermo Scientific).

### Luciferase reporter assays

The 3′-UTRs of the *DNMT1* gene were amplified by PCR from human genomic DNA and cloned into the pMIR reporter vector using the SacІ and HindIII sites immediately downstream from the stop codon of luciferase. The primers were as follows: forward 5′-TCGGAGCTCGGAGGAGGAAGCTGCTAAG-3′ and reverse 5′-GCGAAGCTTTTGGTTTATAGGAGAGAT-3′. We also constructed a mutant report gene using a QuikChange Lightning Multi Site-Directed Mutagenesis Kit (Stratagene Company, La Jolla, San Diego, CA, USA). Each vector, along with 50 nM *miR-377*, was transfected into HEK293 cells with Lipofectamine 2000 reagent (Invitrogen, Carlsbad, CA, USA) according to the manufacturer's instructions. Firefly and renilla luciferase activities were measured consecutively using dual luciferase assays (Promega, Madison, WI, USA) at 48 h after transfection.

### Detection of promoter methylation of senescent genes

Genomic DNA extraction was accomplished using a TIANamp Genomic DNA Kit (TianGen Biotech, Beijing, China) according to the manufacturer's instructions. Bisulfite conversion was carried out using an EZ DNA Methylation-Lightning Kit (ZymoResearch, Irvine, CA, USA), and bisulfite sequencing primer design for each gene was achieved using the online design tool Methprimer (http://www.urogene.org/methprimer/). High-throughput microfluidic PCR for target enrichment, next-generation bisulfite sequencing, sequencing data alignment, and methylation analysis were performed as previously described.^48^

### Statistical analysis

All data were expressed as the mean±standard error of the mean. For comparisons, paired-samples *t* test, a one-way analysis of variance with least significant difference post-hoc tests or analysis of variance for multiple comparisons were performed as appropriate(SPSS Software 19.0). Linear regression analysis (*R*^2^) was used for the correlation analysis. Differences with *P*-values of <0.05 were considered significant.
