# Correction: miR-377 induces senescence in human skin fibroblasts by targeting DNA methyltransferase 1

**DOI:** 10.1038/s41419-019-2097-9

**Published:** 2019-11-12

**Authors:** Hong-fu Xie, Ying-zi Liu, Rui Du, Ben Wang, Meng-ting Chen, Yi-ya Zhang, Zhi-li Deng, Ji Li

**Affiliations:** 1 Department of Dermatology, Xiangya Hospital, Central South University, Changsha, China; 2 Center for Molecular Medicine, Xiangya Hospital, Central South University, Changsha, China; 3 State Key Laboratory of Medical Genetics, Central South University, Changsha, Hunan China; 4 Key Laboratory of Organ injury, Ageing and Regenerative Medicine of Hunan Province, Changsha, China

**Correction to**: **Cell Death and Disease**

10.1038/cddis.2017.75, published online 9 March 2017

Following the publication of this article, the authors noticed an error in Fig. 4a, where the wrong image was used to compile the figure. The correct figure has been provided. The correction does not affect the conclusions of the article. The authors apologise for any inconvenience this may have caused.

**Figure 1:**
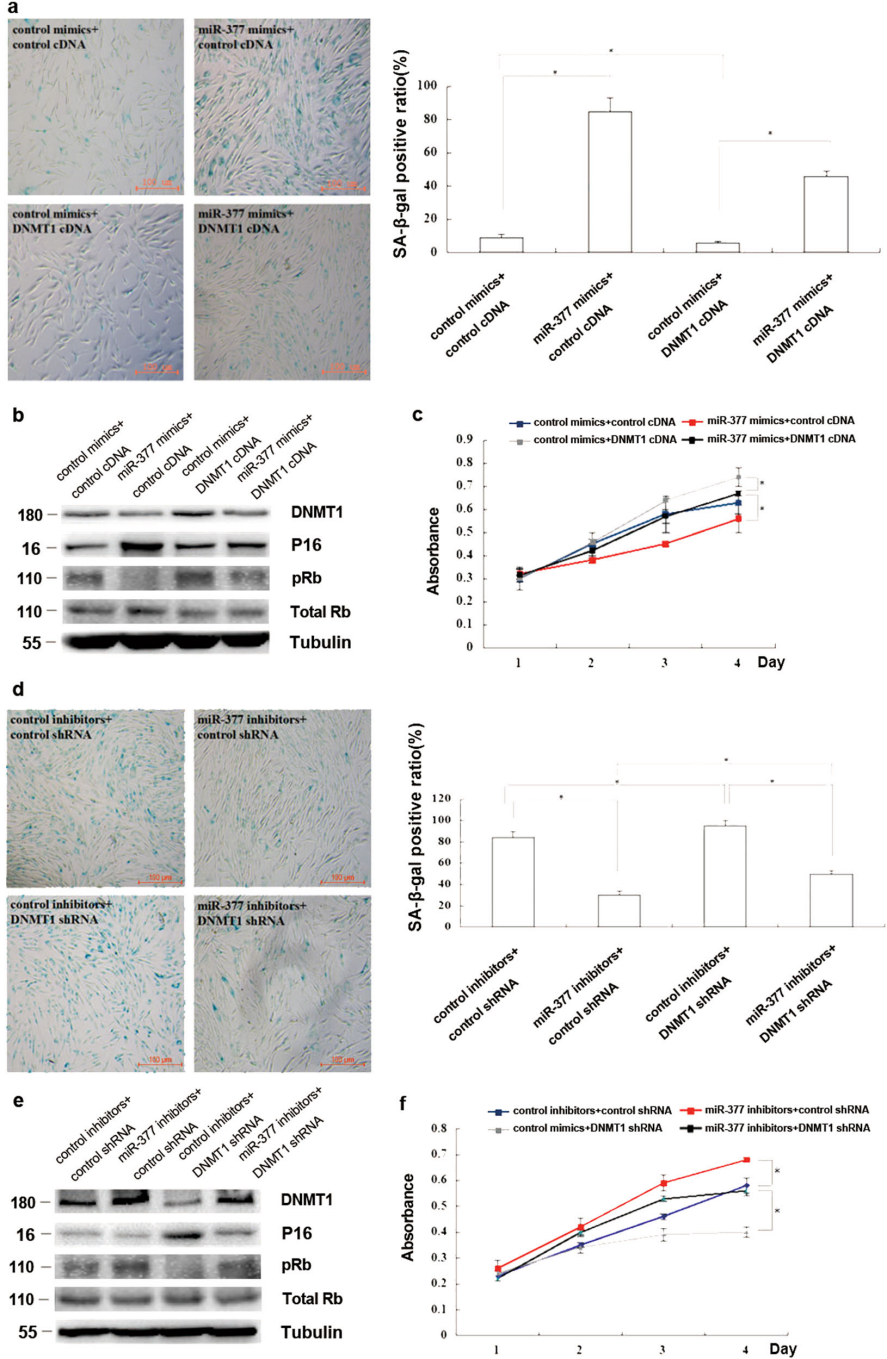
Fig. 4

