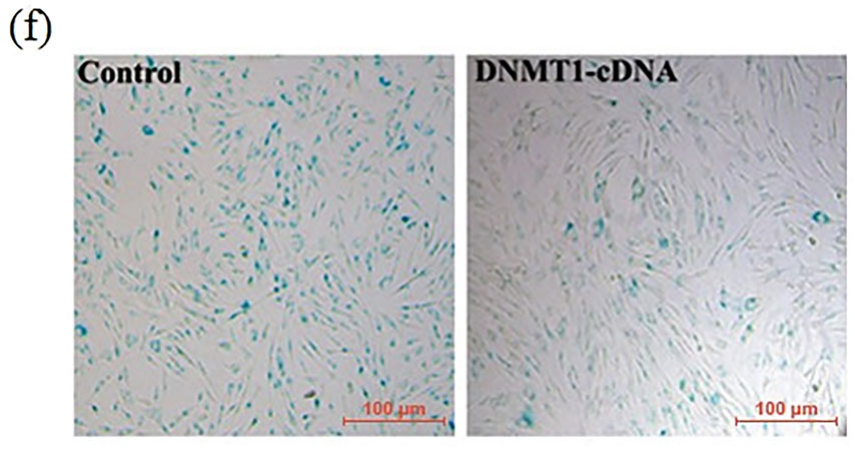# Correction: miR-377 induces senescence in human skin fibroblasts by targeting DNA methyltransferase 1

**DOI:** 10.1038/s41419-025-08184-w

**Published:** 2025-11-17

**Authors:** Hong-fu Xie, Ying-zi Liu, Rui Du, Ben Wang, Meng-ting Chen, Yi-ya Zhang, Zhi-li Deng, Ji Li

**Affiliations:** 1 Department of Dermatology, Xiangya Hospital, Central South University, Changsha, China; 2 Key Laboratory of Organ injury, Ageing and Regenerative Medicine of Hunan Province, Changsha, China; 3 Center for Molecular Medicine, Xiangya Hospital, Central South University, Changsha, China; 4 State Key Laboratory of Medical Genetics, Central South University, Changsha, Hunan China

Correction to: *Cell Death and Disease* 10.1038/cddis.2017.75, published online 9 March 2017

While archiving data from previous publications, the authors identified an error in Figure 1f, where an incorrect image was used in the right panel due to a mislabeling issue. The correct figure has been provided. This correction does not affect the conclusions of the article. The authors apologize for any inconvenience it may have caused.

Original Fig. 1f

**Figure:**
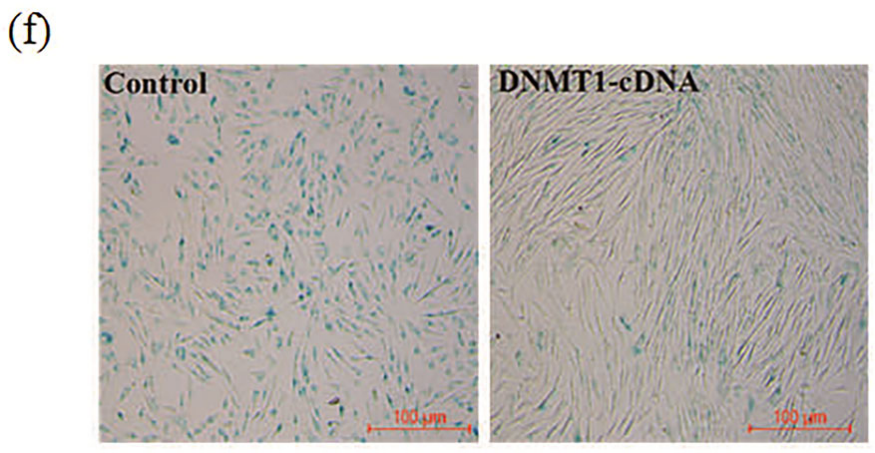

Amended Fig. 1f

**Figure:**