# Supplementary material for: miR-377 induces senescence in human skin fibroblasts by targeting DNA methyltransferase 1
Source: Cell Death Dis. 2017 Mar 9;8(3):e2663–. doi: 10.1038/cddis.2017.75 (PMC5386568; doi:10.1038/cddis.2017.75)
Supplement: Supplementary Information [file cddis201775x1.ppt]

## Slide 1
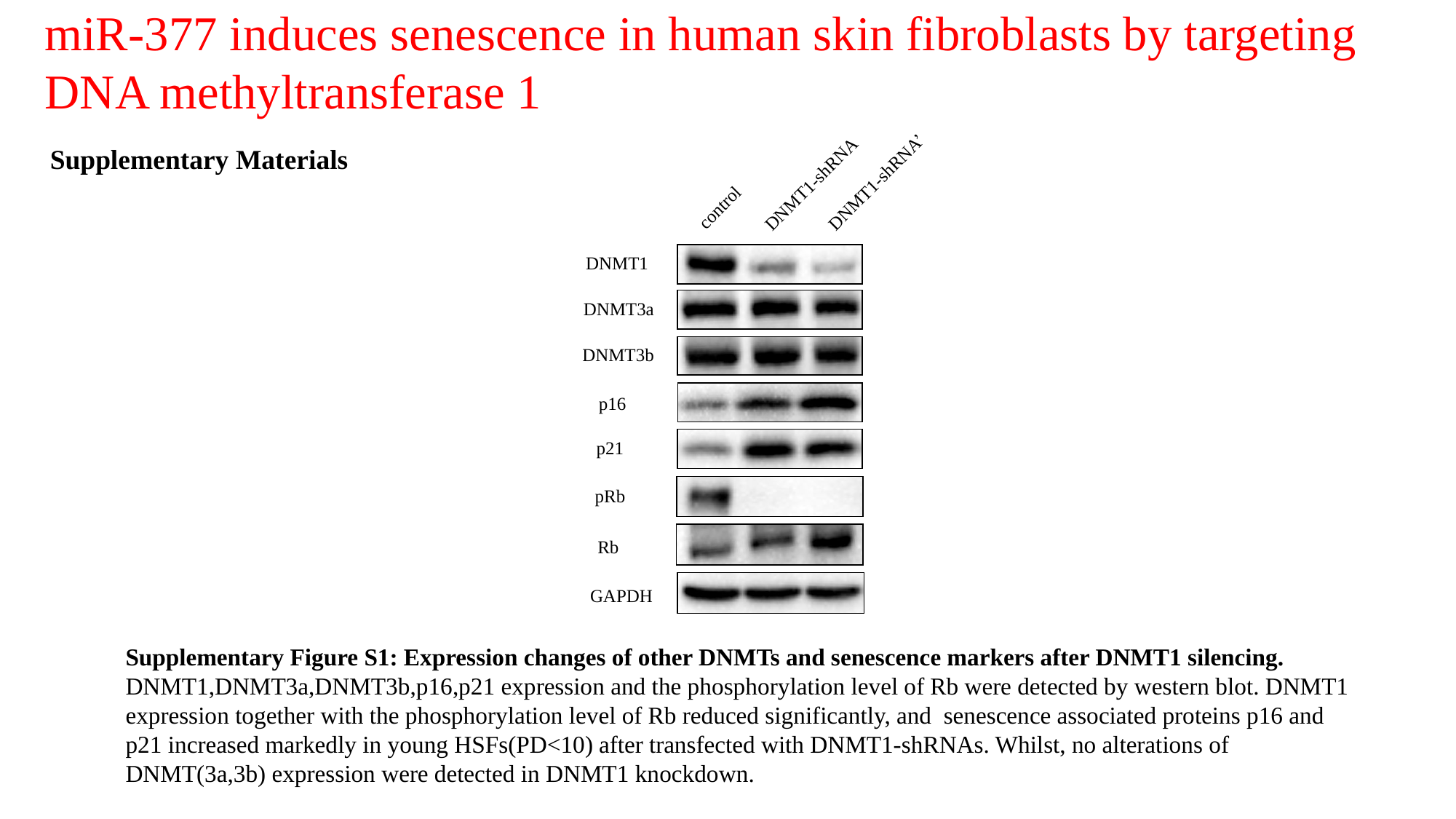

miR-377 induces senescence in human skin fibroblasts by targeting DNA methyltransferase 1
Supplementary Materials
DNMT1-shRNA’
DNMT1-shRNA
control
DNMT1
DNMT3a
DNMT3b
p16
p21
pRb
Rb
GAPDH
Supplementary Figure S1: Expression changes of other DNMTs and senescence markers after DNMT1 silencing. DNMT1,DNMT3a,DNMT3b,p16,p21 expression and the phosphorylation level of Rb were detected by western blot. DNMT1 expression together with the phosphorylation level of Rb reduced significantly, and senescence associated proteins p16 and p21 increased markedly in young HSFs(PD<10) after transfected with DNMT1-shRNAs. Whilst, no alterations of DNMT(3a,3b) expression were detected in DNMT1 knockdown.

## Slide 2
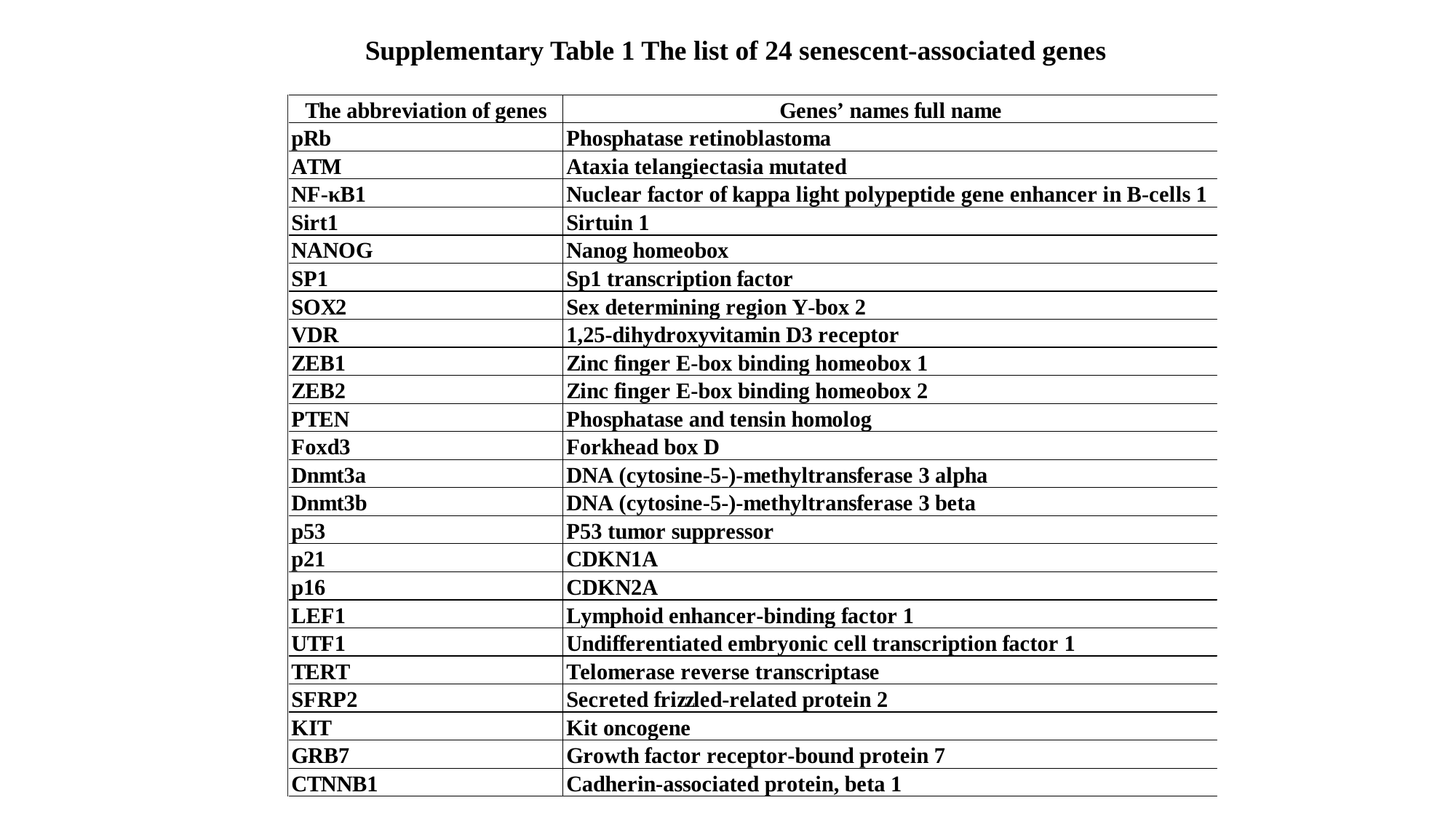

Supplementary Table 1 The list of 24 senescent-associated genes
